# Transcriptome-wide analysis of chromium-stress responsive microRNAs to explore miRNA-mediated regulatory networks in radish (*Raphanus sativus* L.)

**DOI:** 10.1038/srep14024

**Published:** 2015-09-11

**Authors:** Wei Liu, Liang Xu, Yan Wang, Hong Shen, Xianwen Zhu, Keyun Zhang, Yinglong Chen, Rugang Yu, Cecilia Limera, Liwang Liu

**Affiliations:** 1 National Key Laboratory of Crop Genetics and Germplasm Enhancement, College of Horticulture, Nanjing Agricultural University, Nanjing 210095, P.R.China; 2 Department of Plant Sciences, North Dakota State University, Fargo, ND 58108, USA; 3 College of Life Sciences, Nanjing Agricultural University, Nanjing 210095, P.R.China; 4 School of Earth and Environment, and The UWA’s Institute of Agriculture, The University of Western Australia, Perth, WA 6009, Australia

## Abstract

MicroRNAs (miRNAs) are small noncoding RNAs that play pivotal roles in plant growth, development and stress response. Chromium (Cr) is one of common environmental contaminants possessing potential health hazards to living organisms. To date, little is known about the regulatory roles of miRNAs in response to Cr stress in radish. To systematically identify Cr-responsive miRNAs and their targets in radish, two sRNA libraries derived from Cr-free (CK) and Cr-treated (Cr200) roots were constructed. With Solexa sequencing, 81 known and 72 novel miRNAs were identified, from which 54 known and 16 novel miRNAs were significantly differentially expressed under Cr stress. Several target genes for Cr-responsive miRNAs encode different transcription factor (TF) families, including *SPLs*, *MYBs*, *ERFs* and *bZIPs*, might regulate corresponding HM-related transcriptional processes in plants. Notably, a few key responsive enzymes or proteins, including HMA, YSL1 and ABC transporter protein were involved in Cr uptake and homeostasis process. Furthermore, the expression patterns of some Cr-responsive miRNAs and their targets were validated by RT-qPCR. This study represents the first characterization of Cr-responsive miRNAs and their targets in radish. The outcomes of this study could provide novel insights into miRNA-mediated regulatory mechanisms underlying plant response to Cr stress in root vegetable crops.

Chromium (Cr), the seventh most abundant element in the earth’s crust, is one of the most widely distributed environmental contaminants released into the atmosphere mainly due to its huge industrial use^1^. In recent years, environment contamination with Cr has become a main area of concern, especially the hexavalent Cr (Cr^6+^) is an extremely toxic carcinogen^2^,^3^. Phytotoxicity caused by Cr contamination can result in inhibition of seed germination, nutrient balance and enzymatic activities, reduction of root growth and biomass as well as induction of leaf chlorosis and oxidative stress in plants^4^. Moreover, the accumulation of high concentrations of hexavalent Cr in food plants might pose potential health hazards to humans and animals^5^. Although numerous studies have focused on the chemical and physiological characterization of Cr toxicity and tolerance in plants, the complexity of molecular mechanisms underlying the uptake, accumulation, translocation and detoxification in plants remain unknown and it is a challenge in research.

MicroRNAs (miRNAs), approximately 19–24 nucleotides (nt) in length, are a subset of small, endogenous noncoding RNAs which mediate gene expression at the transcriptional and/or posttranscriptional level by targeting mRNAs for degradation or translational repression^6^. In plants, a long primary transcripts of miRNAs are transcribed from a nuclear-encoded miRNA gene and processed into precursors of miRNAs that form secondary stem-loop structures, which are processed by the Dicer-like1 (DCL1) into miRNA:miRNA* duplexes. The mature miRNAs derived from the duplex are subsequently incorporated into the RNA induced silencing complex (RISC) and guide RISC to bind to cognate target transcripts for targeting mRNA cleavage or translational inhibition by sequence complementarity^6^,^7^. Recently, increasing evidences indicate that miRNAs have emerged as effective regulators of numerous abiotic stresses such as nutrition deficiency^8^, cold^9^, salinity^10^, drought^11^, oxidative stress^12^ and heavy metal (HM) stress^13^,^14^.

Recently, with the advent of the next-generation sequencing (NGS) technology, an increasing number of miRNAs have been successfully identified in several vegetable crops, such as Chinese cabbage^15^, tomato^16^, celery^17^ and cucumber^18^. Understanding HM-regulated gene expression and regulatory networks could help clarify the complex molecular genetic mechanisms of metal accumulation and homeostasis^7^. A number of studies have revealed that miRNA-guided gene regulation could play critical roles in plant response to HM stresses^13^,^19^. To date, many studies have demonstrated that miRNAs are hypersensitive to different HMs such as cadmium (Cd), mercury (Hg), aluminum (Al), arsenic (As) and lead (Pb) in some plant species including *Arabidopsis thaliana*, *Oryza sativa*, *Medicago truncatula*, *Populus tomentosa* and *Brassica juncea*^20^. These studies provided valuable information on the involvement of miRNAs in the complex HM responsive networks, however, understanding the miRNA-guided regulatory networks of Cr stress response remains in its infancy.

Radish (*Raphanus sativus* L., 2n = 2x = 18) is an economically important root vegetable crop with an edible taproot of the Brassicaceae family. Since the plant roots were the first vulnerable parts directly exposed to metal-contaminated soils, it’s of vital significance to explore the molecular regulatory networks of HM tolerance and homeostasis in radish root system. Recently, Xu *et al.* identified 15 known and 8 novel Cd stress-regulated miRNA families in radish roots^13^. Some HM-responsive miRNAs have also been identified in some other plant species^19^,^21^,^22^. Srivastava *et al.* detected 69 arsenic (As) stress-induced miRNAs belonging to 18 plant miRNA families in *B. juncea*^19^. Nevertheless, the miRNA-guided regulation networks of Cr stress response in radish need to be clarified. To systematically identify Cr-responsive miRNAs and their targets at the genome-wide level in radish, this study constructed and sequenced two small RNA libraries derived from the Cr-free (CK) and Cr-treated (Cr200) roots using Solexa sequencing technology. The aims of this investigation were to detect Cr-responsive miRNAs and validate the target transcripts for Cr stress-regulated miRNAs in radish. These results would facilitate our understanding of the miRNA-guided regulatory networks of Cr stress in radish, and provide valuable information for further elucidation of molecular mechanisms underlying plant response to Cr stress in root vegetable crops.

## Results

### Overview of transcriptome and sRNA sequencing in radish

To establish a comprehensive reference sequence database, a cDNA library constructed from radish root was sequenced, totally resulting in 51.2 million clean reads. From that, a total of 130,953 contigs and 70,168 unigenes were obtained by trinity assembly [NCBI Sequence Read Archive (SRA) with the GenBank accession No.SRS706782]. These mRNA transcriptome sequences, together with the GSS and EST sequences which have deposited in NCBI databases formed the radish reference sequences for miRNAs identification, as well as the target genes prediction in radish.

Totally, 37.681 million (M) raw reads representing 8.693 M unique sequence reads were generated from the two sRNA libraries (Supplementary Table S1 and Supplementary Fig. S1). Deep sequencing of the two libraries (CK and Cr200) generated 18.473 M and 20.530 M raw reads, respectively (Supplementary Table S2). Based on removal of the low-quality tags and corrupted adapter sequences, 18,127,561 (representing 3,360,437 unique sequences) and 19,552,260 (representing 6,172,992 unique sequences) clean reads were obtained from CK and Cr200 library, respectively (Supplementary Table S2 and S3). After removing the noncoding sRNAs including rRNAs, snRNAs, snoRNAs and tRNAs, the remaining sequences were queried against the miRBase 21.0 database. From that, 1,680,180 (CK) and 2,057,888 (Cr200) total sRNA sequences were found to be similar to known miRNAs (Supplementary Table S3). The remaining 14,929,650 (CK) and 14,626,333 (Cr200) unannotated total sRNA sequences were used for the identification of novel miRNAs (Table 1).

**Figure 1:**
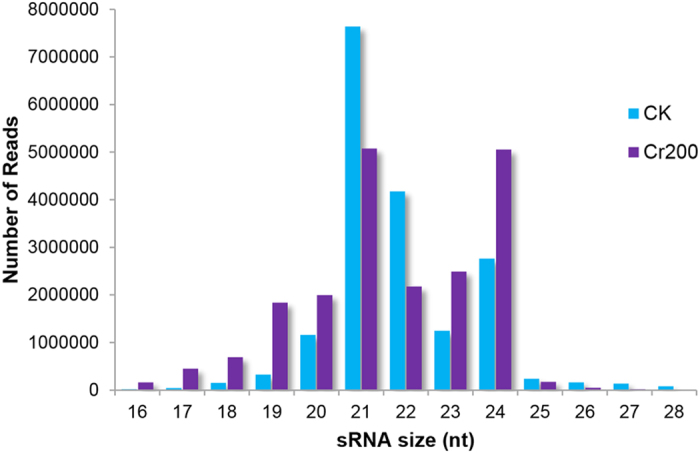
Size distribution of small RNAs in CK and Cr200 libraries from radish roots.

The length distribution of sRNA sequences ranged from 16 to 28 nt with the 21-nt sRNAs represented the most frequent length in both two libraries (Fig. 1). Among these small RNAs, both 21- and 22-nt sequences in the CK library were notably higher than those in the Cr200 library, indicating that both two kinds of length of miRNAs might be inhibited under Cr stress. Conversely, the abundance of 24-nt sRNAs in the CK library was lower than that in the Cr200 library, implying that the biogenesis of the 24-nt sRNAs may be highly relevant to Cr stress.

### Identification of the known miRNAs in radish

**Figure 2:**
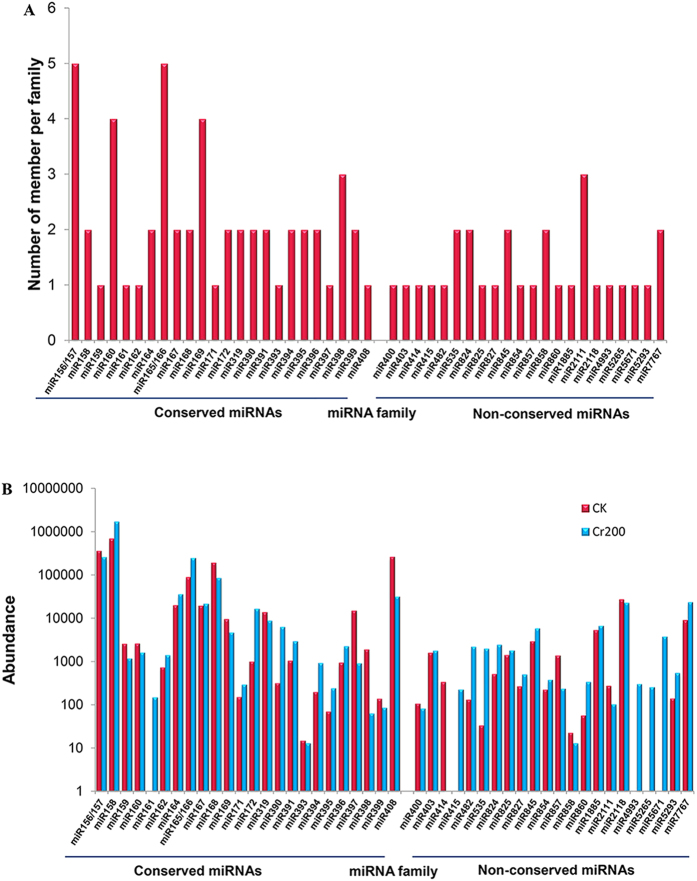
Number and abundance of identified known miRNA families from radish. (**A**) Distribution of known miRNA family number. (**B**) Count of each known miRNA family.

From the identified 20,156 (CK) and 43,980 (Cr200) known individual candidate miRNAs (Table 1), the conserved and non-conserved miRNAs were filtered by aligning against miRBase 21.0, allowing no more than two mismatches. Ultimately, 52 known miRNAs belonging to 24 conserved families were identified in both libraries (Table 2 and Supplementary Table S4). The number of members for conserved miRNA families differs dramatically in both two libraries (Table 2 and Fig. 2A). The miR169 as well as miR156/157 and miR165/166 possessed four and five members, respectively; while seven miRNA families (miR159, miR161, miR162, miR171, miR393, miR397 and miR408) each had only one member. Moreover, the counts of reads for different known miRNA families exhibited large divergences. A few conserved miRNA families such as miR156/157, miR158, miR165/166, miR168 and miR408 showed extraordinarily high expression abundance in both the CK and Cr200 libraries. The miR158 family exhibited the most abundant expression level, with 699,489 (CK) and 1,730,976 (Cr200) reads accounting for 40.86% and 69.97% of all conserved miRNA reads, respectively (Fig. 2B). Several miRNA families including miR164, miR167 and miR319 were moderately abundant in the two libraries.

In total, 29 unique sequences representing 22 non-conserved miRNA families were also discovered in the two libraries. A large number of non-conserved miRNA families (eg. miR400, miR825, miR827, miR854, miR860, miR1885, etc) contained only one member and they had a moderate or low abundance in the two libraries; whereas five miRNA families (miR535, miR824, miR845, miR858 and miR7767) and miR2111 comprised two and three members, respectively (Table 2 and Supplementary Table S4), indicating that the non-conserved miRNAs had comparatively lower abundance than the conserved miRNAs. Interestingly, the number of reads for different miRNA members belonging to one known miRNA family varied drastically. For instance, the normalized read counts of miR535 varied from 0.01 to 104.0289 (Supplementary Table S4), which revealed large variation in the expression abundance of known miRNA families in radish.

### Identification of novel candidate miRNAs in radish

The criteria, the characteristic stem-loop precursor, were selected to annotate novel miRNAs^23^. Based on the miRNA:miRNA* criterion, 72 novel miRNA sequences belonging to 54 miRNA families were identified as putative novel miRNAs, of which six were detected to have complementary miRNA*s (Supplementary Table S5). The length of these novel miRNAs varied from 20 to 23 nt, with the majority being 21 nt (Supplementary Fig. S2). The mean length of the novel miRNA precursors was 154 nt with a range of 47 to 332 nt. The stem loop structures of these predicted novel miRNA precursors were shown in Supplementary Fig. S3. Furthermore, the values of minimum folding free energy (MFE) ranged from −118.5 to −18.3 kcal mol^−1^ with an average of −49.9 kcal mol^−1^. The expression levels of novel miRNAs varied significantly between the two libraries, and 21 and 42 miRNAs were only detected in CK and Cr200 library, respectively (Supplementary Table S5), which may be attributed to the fact that the expressions of some miRNAs are specifically repressed by Cr stress.

### Differentially expressed miRNAs in response to Cr in radish

To systematically identify Cr-responsive miRNAs in radish, a differential expression pattern analysis was performed between the two libraries. In total, 54 known and 16 potentially novel miRNAs were identified to be differentially expressed under Cr stress (Tables 3 and 4). Of these, 37 miRNAs (28 known and nine novel miRNAs) were induced. Among the 33 down-regulated miRNAs (26 known and seven novel miRNAs) under Cr stress, miR169r-3p and rsa-miRn-2 were the most significantly down-regulated known (fold change −15.57) and novel (fold change −12.78) miRNA, respectively. The majority of these differentially expressed novel miRNAs (eg. rsa-miRn1, rsa-miRn2, rsa-miRn10, rsa-miRn12, rsa-miRn14, rsa-miRn19, rsa-miRn23, rsa-miRn28 and rsa-miRn44) exhibited significant differences in expression profiles. For instance, rsa-miRn1, rsa-miRn2, rsa-miRn10, rsa-miRn14 and rsa-miRn19 were only discovered in the CK library, whereas rsa-miRn23, rsa-miRn28, rsa-miRn44, rsa-miRn45 and rsa-miRn54 were only detected in the Cr200 library (Table 4). These significant variations in miRNA expression implied critical roles of these differentially expressed miRNAs in Cr stress response in radish.

### Prediction and annotation of putative miRNA targets in radish

**Figure 3:**
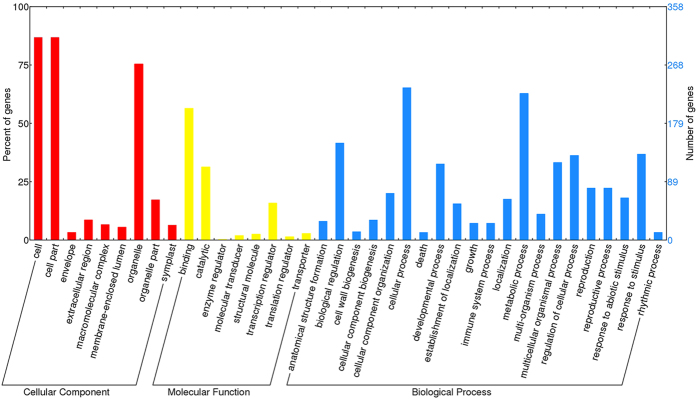
Gene ontology classification of target transcripts for all differentially expressed miRNAs in radish.

**Figure 4:**
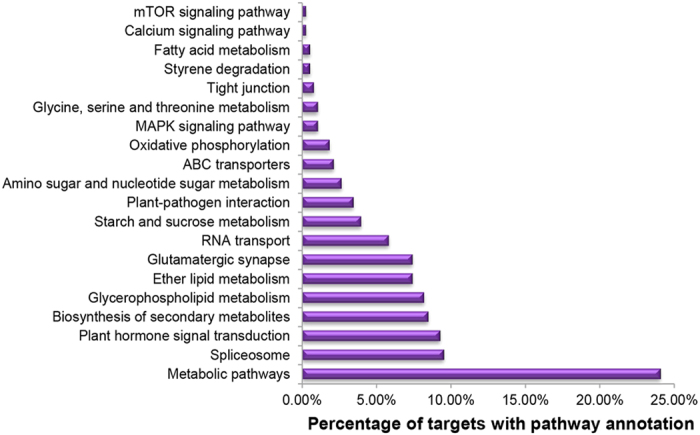
The most enriched KEGG pathways of target genes for differentially expressed miRNAs.

The prediction of the targets for miRNAs is a prerequisite to thoroughly elucidate the molecular functions of the Cr-responsive miRNAs. In this study, 358 genes were predicted to be targets for 70 Cr-responsive miRNAs (Table 5 and Supplementary Table S6). All target transcript sequences were successfully classified into three GO terms including biological processes, molecular functions and cellular components using Blast2GO program (Supplementary Table S6). Among the GO terms, the predominant terms involved in biological processes were ‘cellular process’ (GO:0009987), ‘metabolic process’ (GO:0008152), ‘biological regulation’ (GO:0065007) and ‘response to stimulus’ (GO:0050896), while ‘binding’ (GO:0005488), ‘catalytic activity’ (GO:0003824) and ‘transcription regulator activity’ (GO:0030528) were the most abundant terms in molecular functions. For cellular components, those assignments were mostly given to ‘cell’ (GO:0005623), ‘cell part’ (GO:0044464) and ‘organelle’ (GO:0043226) (Fig. 3). Moreover, a total of 180 KEGG pathways were enriched for the target genes of differentially expressed miRNAs (Supplementary Table S7). The categories of metabolic pathways [ko01100], spliceosome [ko03040], plant hormone signal transduction [ko04075], biosynthesis of secondary metabolites [ko01110] and glycerophospholipid metabolism [ko00564] were among the most enriched pathways (Fig. 4). Among them, the calcium signaling pathway [ko04020], MAPK (mitogen-activated protein kinase) signaling pathway [ko04010], ABC transporters [ko02010] as well as glycine, serine and threonine metabolism [ko00260] play important roles in response to various HMs.

Moreover, the miRNA targets in response to Cr stress in radish were further investigated to better elaborate the functions of these target genes. Analyses on 37 and 14 targets identified 19 known and six novel miRNA families, respectively (Table 5 and Supplementary Table S8). The prediction details of other unclassified miRNAs are shown in Supplementary Table S9. The results indicated that many of the identified target genes for the known miRNAs encoded transcription factors, such as myb domain proteins (*MYBs*), auxin response factors (*ARFs*), NAC domain transcription factors (*NACs*), ethylene-responsive transcription factors *(ERFs*), basic leucine zippers (*bZIPs*), basic Helix-Loop-Helix proteins (*bHLHs*), squamosa promoter-binding-like proteins (*SPL*s), *TCPs* and *WRKYs* (Supplementary Table S8), which were involved in the processes of plant growth and development.

A few transcripts were annotated to genes involved in response to biotic and abiotic stresses. For instance, miR160 targeted aluminum-activated malate transporter 9 gene (*ALMT9*), miR164 regulated ERF073 gene (*HRE1*), miR825 cleaved TIR-NBS-LRR disease resistance gene and miR4993 regulated gene encoding HM transport/detoxification domain-containing protein. Additionally, several target genes encoded some important enzymes or functional proteins such as laccase, DEAD-box ATP-dependent RNA helicase, putative galacturonosyltransferase-like (*GATL*), translation initiation factor (TIF), cytochrome P450 and yellow stripe-like 1 transporter (*YSL1*), which could play indispensable roles in diverse metabolic pathways. In general, gene annotation indicated that most of these identified target genes were involved in various signal sensing and transduction and related secondary metabolite processes, such as ROS (reactive oxygen species) signaling, organic acid biosynthesis, cell wall synthesis, brassinosteroid biosynthesis and the biosynthesis of jasmonic acid (JA), which were considered to be HM-stress related transcripts activated in response to HMs.

### RT-qPCR validation

**Figure 5:**
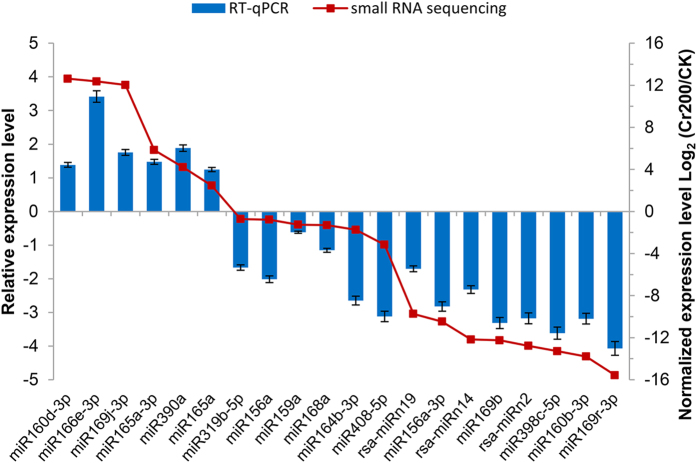
RT-qPCR validation of differentially expressed miRNAs under Cr stress in radish. Each bar shows the mean ± SE of triplicate assays.

To validate the expression profiles of the differentially expressed miRNAs identified from the high-throughput sequencing, a total of 20 Cr-responsive miRNAs were randomly selected for quantitative RT-PCR assays. The expression patterns of the selected miRNAs shared a similar tendency of the correlation between the RT-qPCR and the small RNA sequencing (Fig. 5). The fold changes of some miRNAs from the deep sequencing were relatively higher than those obtained from the RT-qPCR analysis, which partially attributed to the differences in the sensitivity, specificity and algorithm between the two techniques. The small RNA sequencing generated absolute expression patterns while relative expressions for RT-qPCR analysis.

**Figure 6:**
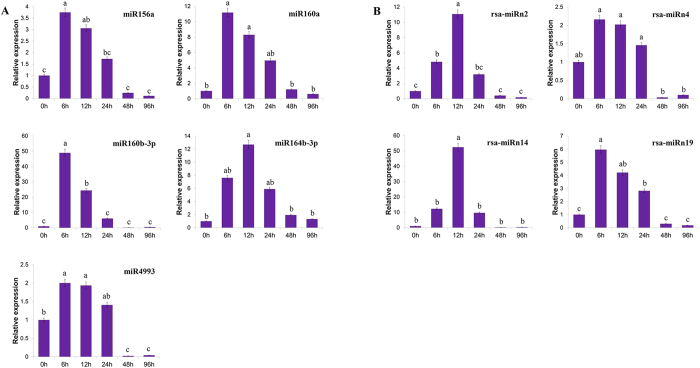
RT-qPCR validation of Cr-responsive known (A) and novel (B) miRNAs in radish. Each bar shows the mean ± SE of triplicate assays. The values with different letters indicate significant differences at *P* < 0.05 according to Duncan’s multiple range tests.

**Figure 7:**
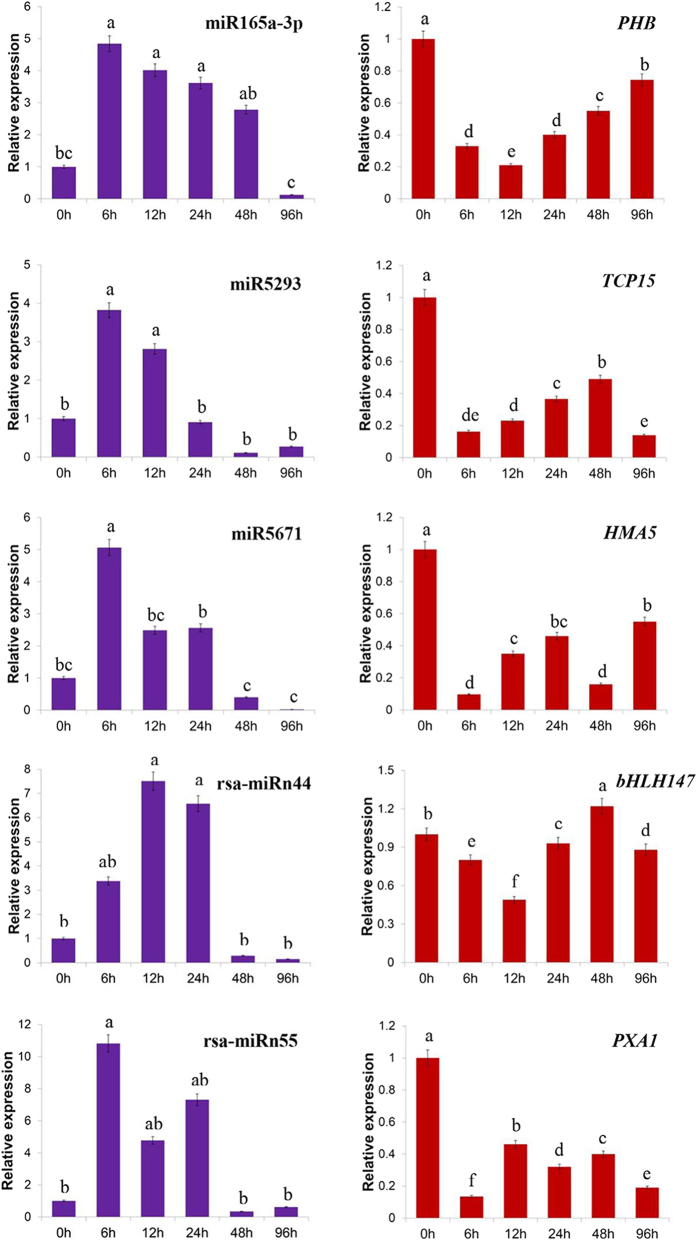
RT-qPCR validation of Cr-responsive miRNAs and their target genes in radish. *PHB*, *TCP15*, *HMA5*, *PXA1* and *bHLH147* represent genes encoding Homeobox-leucine zipper protein ATHB-14, Transcription factor TCP15, putative Copper-transporting ATPase HMA5, ABC transporter D family member 1 and Transcription factor bHLH147, respectively. Each bar shows the mean ± SE of triplicate assays. The values with different letters indicate significant differences at *P* < 0.05 according to Duncan’s multiple range tests.

In addition, randomly selected eight known and six novel miRNAs were subjected to RT-qPCR analysis to investigate the dynamic expression profiles of the Cr-responsive miRNAs in radish plants exposed to various durations to Cr (eg. 0, 6, 12, 24, 48 and 96 h). As shown in Figs 6 and 7, four miRNAs (miR156a, miR160a, miR165a-3p and rsa-miRn19) and three miRNAs (miR164b-3p, rsa-miRn2 and rsa-miRn44) were up-regulated and maximized at 6 h and 12 h, respectively, and then gradually declined during other exposure time points. Similar expression pattern was also evidenced for miR160b-3p, miR4993, miR5293 and rsa-miRn4 with the greatest expression at 6 h, and then dramatically dropped to a relative low level at 48 h and 96 h. Transcripts of miR5671 and rsa-miRn55 had an up-regulated expression pattern which was peaked at 6 h and then sharply declined at later stages. Similarly, expression of rsa-miRn14 was sharply increased and reached its maximum at 12 h, then abruptly decreased and remained at a relative low level after 48 h and 96 h exposure to Cr.

To further confirm the dynamic correlation between the miRNAs and their corresponding targets under Cr stresses, the expression patterns of five predicted target genes including *PHB* (CL6137.Contig2 targeted by miR165a-3p), *TCP15* (Rsa#S42010504 targeted by miR5293), *HMA5* (Unigene10987 targeted by miR5671), *PXA1* (CL5442.Contig2 targeted by rsa-miRn55) and *bHLH147* (Rsa#S42032440 targeted by rsa-miRn44) were examined by RT-qPCR at different Cr-treated time points (0, 6, 12, 24, 48 and 96 h). An approximate negative correlation was observed between the expression of Cr-responsive miRNAs and their targets. For instance, miR165a-3p, miR5293 and rsa-miRn44 as well as their corresponding target transcripts exhibited contrary expression tendencies during the treatment stages (Fig. 7). Overall, the results revealed that some miRNAs might play crucial roles in plant response to HM stress by negatively regulating their corresponding targets in radish.

## Discussion

Pollution of soils by heavy metals (HMs) has becoming an ever-growing problem throughout the world^24^. Chromium is known to be a toxic metal potentially threatening the health of plants and consequently human beings^5^. Plant responses to metal toxicity exhibit various physiological and biochemical processes that require fine and precise regulation at transcriptional and posttranscriptional levels^25^. Recently, a number of miRNAs and their corresponding targets have been comprehensively identified using high-throughput Solexa sequencing technology in some important plant species^7^,^26^,^27^, which proved to be involved in plant response to HM stresses including Cd^13^,^28^, Al^21^,^22^, As^19^, Hg^14^ and Pb^29^. However, few studies on extensive identification of Cr-responsive miRNAs and their target genes have yet been reported in vegetable crops.

### Characteristics of Cr-responsive miRNAs in radish

The identification of a comprehensive set of Cr-responsive miRNAs is an indispensable step to facilitate our understanding of miRNA-guided molecular regulatory mechanisms of plant response to Cr stress. In the present study, a total of 52 conserved and 29 non-conserved miRNAs were successfully identified from the CK and Cr200 libraries. The majority of conserved miRNAs exhibited relatively higher reads compared with the non-conserved counterparts. Moreover, the average member number for conserved miRNA families was larger than that for non-conserved miRNAs, which was in accordance with previous studies in other species such as *B. napus*^28^, *M. truncaula*^21^ and strawberry^30^. Similar to the observation reported previously, most targets for the miRNAs identified in our study were associated with growth and developmental processes or transcription regulation, and fewer were involved in signal transduction or biotic and abiotic stress responses^28^.

In total, 54 known and 16 novel miRNAs were significantly differentially expressed under Cr stress in our study. The majority of these differentially regulated miRNAs were up-regulated, while others were repressed under Cr exposure. Previous reports revealed that the expression of some conserved miRNA families (such as miR159, miR160 and miR319) were down-regulated by most metals, especially the expression of miR159 was always repressed under the HMs exposure^31^. In the current study, in addition to these three miRNAs, some other conserved miRNA families (such as miR156, miR168, miR169, miR397, miR398, miR399 and miR408) were also down-regulated under Cr stress. The families of miR156, miR393 and miR395 were induced in response to most metals previously studied^31^. In our study, the expression of four conserved miRNA families including miR161, miR172, miR390 and miR394, however, was up-regulated under Cr exposure. Furthermore, we found that several reported miRNA families (miR162, miR167, miR171, miR393, miR395 and miR396) did not change significantly in response to Cr stress in radish, although some of them were reported to show differential expression in response to most HMs^31^. In addition, there were some other non-conserved miRNAs (such as miR414, miR857, miR2111, miR4993, etc) that were differentially expressed by Cr stress. It was reported that some previously reported HM-responsive miRNAs showed temporal time-, organ- and species-specific expression patterns. Srivastava *et al.* demonstrated that some As-regulated miRNAs (eg. miR156, miR162, miR165, miR167, etc) showed adverse expression patterns under As (V) stress for 0, 1 and 4 h in *B. juncea*^19^. In this study, different members of these miRNA families showed variable expression under Cr stress. The identification and analysis of miRNAs responsive to different metal toxicity have provided information about their possible relations in the networks involved in plant adaptation to HM stresses. However, further studies are still needed to deeply profile the differential expression patterns and validate the precise regulatory roles of these Cr-responsive miRNAs in radish.

A group of miRNA families (such as miR158, miR162, miR164, miR169, miR319 and miR391) were expressed differentially in shoots and roots^28^. In different plant species, some miRNAs show differential expression even though they were exposed to the same HM. For instance, miR164 and miR172 were down-regulated in *O. sativa*, whilst up-regulated in *B. juncea* when exposed to As stress^19^,^32^. Similarly, miR159, miR162 and miR396 were repressed by AlCl_3_ treatments in *M. truncatula*, whereas these miRNA families were substantially elevated in *N. tabacum* exposed to Al_2_O_3_ nanoparticles^21^,^33^. These phenomena might be caused by the differences in the genetic constitution and tolerance mechanisms between the studied crops.

### TFs and signal transduction involved in Cr stress response in radish

**Figure 8:**
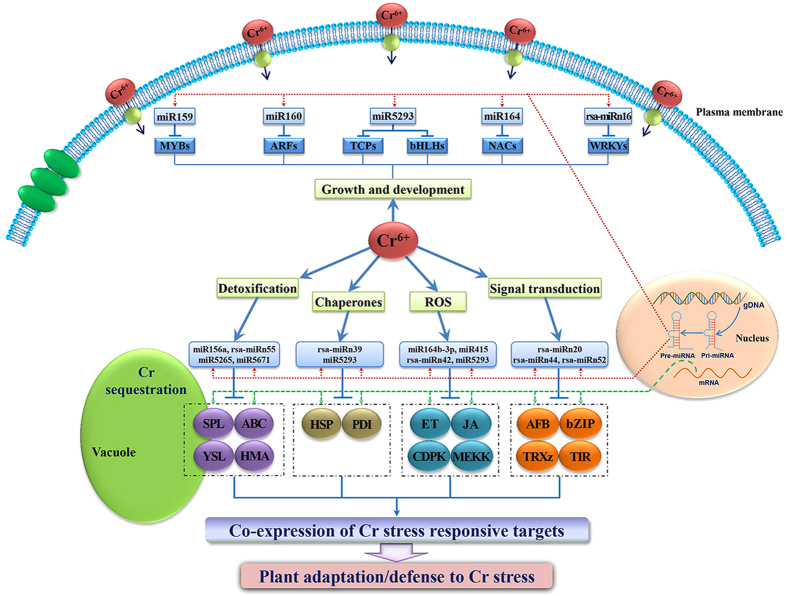
The hypothetical model of regulatory networks of Cr-responsive miRNAs and their target genes in radish. Myb domain protein (MYB), Auxin response factor (ARF), TCP family transcription factor (TCP), basic Helix-Loop-Helix transcription factor (bHLH), NAC domain transcription factor (NAC), WRKY transcription factor (WRKY), Reactive oxygen species (ROS), Squamosa promoter-binding-like protein (SPL), ATP-binding cassette (ABC), Yellow stripe-like (YSL), Heavy metal ATPase (HMA), Heat shock protein (HSP), protein disulfide isomerase (PDI), ethylene (ET), jasmonic acid (JA), calcium-dependent protein kinase (CDPK), mitogen-activated protein kinase kinase kinase (MEKK), Auxin signaling F-box protein (AFB), basic leucine-zipper (bZIP), Thioredoxin-like protein CITRX (TRXz), protein TRANSPORT INHIBITOR RESPONSE (TIR).

Numerous transcription factor (TF) families have been proved to play vital regulatory roles in mediating the expression profiles of HM stress-responsive genes^34^,^35^. In the current study, several identified key targets belonged to a variety of transcription factor families, such as *MYBs*, *SPLs*, *ERFs*, *bZIPs* and *TCPs*, which could regulate corresponding HM-related transcriptional processes in plants^36^ (Fig. 8). Previous studies have revealed that SPL transcription factors targeted by miR156 in *Arabidopsis* were involved in a broad range of developmental and stress response processes including flowering^37^, shoot maturation^38^ and metal homeostasis^39^. For instance, the *SPL* gene family was reported as metal-containing transcription factors regulating Cu homeostasis in *Arabidopsis*^39^,^40^. In this study, three miRNA families (miR156/157, miR159 and miR5293) targeted *SPL3*, *SPL6*, *SPL9*, *SPL13* and *SPL15* belonging to five different classes of the *SPL* gene family. It could be inferred that the miR156/157, miR159 and miR5293 may be vital regulators in Cr^6+^ homeostasis in radish by targeting *SPLs*. In *T. caerulescens*, the R2R3-Myb TF was expressed abundantly under high concentration of Cd^41^. In our study, the MYB family (*MYB3*, *13*, *101*, *104* and *305*) targeted by miR159, miR319 and miR858 might play significant roles in mediating the expression profiles of Cr stress-related genes. Together, the involvement of different TF families could mediate differential expression of some HM responsive genes and transporters responsible for miRNA-mediated Cr stress-responsive regulatory networks to alleviate Cr stress toxicity in radish (Fig. 8).

Signal transduction pathways, which represent important participants in the regulatory networks of plant response to HM stresses, consist of several signaling proteins including calcium-binding related proteins and mitogen-activated protein kinases (MAPKs). In our study, one Ca^2+^-mediated signal-related gene (*CDPK6* targeted by rsa-miRn42) and one transcript encoding *MEKK1* belonging to MAPK family (targeted by miR5293) were identified. Moreover, there were several studies showed that HM stresses (such as Cd, Pb and As) can activate the biosynthesis and accumulation of jasmonic acid (JA)^42^,^43^. Previous studies have proved that TCP transcription factors could bind the TCP-recognized motif (GGACCAC) in the promoter of lipoxygenase (LOX) and regulate the JA biosynthetic pathway^42^. The present study identified a series of TCP genes (*TCP6*, *TCP15* and *TCP21*) for the Cr-responsive miR5293 in radish. These results indicated that the miRNA-mediated signal transduction pathways could regulate the complex networks of Cr^6+^ uptake, transport and gene regulation.

### miRNA-mediated Cr stress-responsive regulatory networks in radish

The characterization of potential targets for stress related miRNAs is a critical step for clarification of miRNA-mediated regulatory networks associated with plant response to HM stresses^29^,^34^. Recently, some studies have been conducted on the identification of various targets for HM-responsive miRNAs, which made great contributions to the understanding of the critical involvement of miRNAs and their target genes in HM toxicity responses in plants^20^,^34^. In this study, a number of key HM-responsive enzymes or proteins mediating the HM uptake, accumulation, translocation and detoxification processes in plants, were identified as target transcripts for several known and novel miRNAs. The yellow stripe-like 1 gene (*YSL1*) was cleaved by miR5265 and could play a pivotal role in HM homeostasis in plants, which functioned as a metal-nicotianamine (NA) transporter for metal ion transport^29^,^44^. In addition, five genes encoding two ABC transporter proteins (PDR5 and PXA1), one heavy metal ATPase (HMA5) and two HM transport/detoxification domain-containing proteins, which were considered as indispensible ingredients in metal uptake and translocation in plants^13^,^28^, were identified for miR7767-3p, rsa-miRn55, miR5671, rsa-miRn44 and miR4993, respectively. TIR-NBS-LRR gene targeted by miR825 is likely to take part in a number of pathways regulating plant response to pathogen attack, and the pathogen resistance proteins may be involved in HM toxicity^14^,^21^,^22^. Laccase was associated with lignification and thickening of the plant cell wall^45^, which may be involved in the reinforcement of the tissue cohesion and in the sequestration of HM ions^46^. In our study, two kinds of laccase genes were identified as the targets of miR397a and rsa-miRn35, respectively. Heat shock proteins (HSPs) have been proved to be expressed in response to HM stresses^47^. In this study, *HSP81-2* gene was identified as the target of rsa-miRn39, indicating that HSPs played critical roles in radish tolerance to Cr stress. Taken together, these genes encoding YSL1, CDPK6 (calcium-dependent protein kinase 6), MEKK1 (mitogen-activated protein kinase kinase kinase 1), HSPs, TIR-NBS-LRRs, laccases, TFs, ABC transporter proteins, HMA and HM transport/detoxification domain-containing proteins, which were identified to be targeted by miRNAs in this study, might play crucial roles in the regulatory networks responsive to Cr stress in radish (Fig. 8).

Based on the comprehensive identification of Cr-responsive miRNAs and analysis of their corresponding target genes, a schematic model of tolerance mechanism and regulatory networks associated with Cr stress response in radish was proposed (Fig. 8). After entry into the plant cell by the actions of several Iron transporter-like proteins, excessive reactive oxygen species (ROS) was generated. Meanwhile, Cr^6+^ could activate the detoxification mechanisms and signaling molecules as well as chaperones, which could eventually enhance the level of adaptation and/or tolerance to Cr stress. After sensing the Cr^6+^ ions, the plant cells could activated some HM stress responsive hormones and signaling molecules (eg. ethylene, JA, CDPK, MEKK, Auxin signaling F-box protein, Thioredoxin-like protein, etc) to decrease Cr^6+^-induced excess ROS level and oxidative damage (Fig. 8). Moreover, some metal transporters such as miR156-targeted SPL, rsa-miRn55- and miR7767-cleaved ABCs, miR5265-regulated YSL and miR5671-mediated HMA were activated to transport Cr^6+^ out of the cell and/or translocate them into the vacuole. In addition, miR5293- and rsa-miRn39-targeted HSPs, known as molecular chaperones functioning in protein folding, reassembly, translocation and degradation, could be involved in repairing damages caused by HM stresses^48^ (Fig. 8). In summary, the co-expression of Cr stress responsive genes could enhance the level of defense or tolerance to Cr stress and alleviate the phytotoxicity of Cr^6+^ in radish.

In conclusion, small RNA sequencing technology and bioinformatics approaches were firstly employed to identify the miRNAs and their target genes in response to Cr stress in radish. In total, 54 known and 16 novel miRNAs were significantly differentially expressed in radish roots under Cr stress. These miRNAs might be involved in various biological processes to alleviate the phytotoxicity and enhance plant tolerance to Cr stress. A large number of the target genes for Cr-responsive miRNAs were mainly involved in stress-related signal sensing and transduction, antioxidant defense, detoxification and homeostasis processes. In addition, the expression profiles of a set of differentially regulated miRNAs and their targets were validated by RT-qPCR assays. These results represent a fundamental step towards elucidating miRNA-mediated regulatory networks and molecular genetic mechanisms underlying plant response to Cr stress.

## Methods

### Plant materials and Cr stress treatment

The seeds of a radish advanced inbred line, ‘NAU-YH’, were surface-sterilized in 1.5% NaClO for 3 min followed by washing and soaking in sterile distilled water. After germination at 25 °C in the dark for 3 days, the seeds were transferred into the plastic pots in a growth chamber (25/18 °C day/night, 16/8 h light/dark). Seedlings with four fully expanded true leaves were transferred into modified half-strength Hoagland’s nutrient solution as described previously^49^. One week later, the plants were treated with 200 mg L^−1^ Cr (VI) for 6, 12, 24, 48 and 96 h, respectively. Seedlings cultured in Cr-free solution were used as control. After the treatments, the plants were separately harvested and frozen immediately in liquid nitrogen and stored at −80 °C for further use. To minimize biological difference, root samples from three replicates were equally pooled. The roots grown in the Cr-free solution (control) and those treated with Cr (VI) for 48 h were used to construct control (CK) and Cr-treated (Cr200) sRNA library, respectively.

### Small RNA (sRNA) library construction and sequencing

Total RNA was extracted from Cr-free (CK) and Cr-treated (Cr200) radish roots using TRIzol Reagent (Invitrogen, USA) following the manufacturer’s instructions. Two sRNA libraries were constructed according to the reported procedures^13^,^49^. Briefly, the small RNA fragments ranging from 18–30 nt were isolated, purified and subsequently ligated to Solexa adapters at each end. Then the assembled small RNAs were reverse transcribed to cDNA and small RNA libraries were finally sequenced with Solexa sequencing (Illumina) at the Beijing Genomics Institute (BGI, China).

### Analysis of small RNA sequencing data

Small RNA reads were obtained from Illumina HiSeq™ analyzer. The clean reads were screened from raw data by filtering out the corrupted adapter sequences (such as 3′ adapter_null, insert_null and 5′ adapter_contaminants), poly-A tails and sequences with ≤18 nt and ≥30 nt. Then the unique sequences were aligned with the radish reference sequences including the radish transcriptome sequences [NCBI Sequence Read Archive (SRA) with the GenBank accession No.SRS706782], expressed sequence tag (EST) sequences and genomic survey sequences (GSS) using SOAP2 program^50^. Perfectly matched sequences were used for subsequent analysis. Reads mapped to known noncoding RNA families including rRNAs, tRNAs, snRNAs and snoRNAs in the Rfam 12.0 (http://rfam.xfam.org/) and NCBI GenBank (http://www.ncbi.nlm.nih.gov/genbank/) databases were excluded from further analysis. Then the remaining mapped sequences were aligned against miRBase 21.0 (http://www.mirbase.org/index.shtml) to identify known miRNAs with no more than two mismatches. Thereafter, the unannotated unique sequences were used for novel miRNAs prediction according to the criteria of novel miRNAs^23^ using Mireap software (https://sourceforge.net/projects/mireap/). Secondary structures of candidate miRNA precursors were confirmed by Mfold software^51^.

### Differential expression analysis of miRNAs responsive to Cr stress

The expression abundance of miRNAs in the two libraries was normalized to one million by total clean reads of miRNAs per sample (normalized expression = actual miRNA count/total count of clean reads ×1,000,000)^13^. Following the normalization, if the abundance of a given miRNA is zero, the expression value was set to 0.01; if the normalized miRNA reads were less than 1 in both libraries, they were not used for differential expression analysis owing to their too low abundance. The fold change between the two libraries was calculated as: fold change = log_2_ (Cr200/CK). The *P*-value was calculated based on previously established methods^52^. The values of log_2_ (Cr200/CK) ≥ 2 or <−0.5, along with the *P* ≤ 0.05, were used as the thresholds to judge the up-regulated or down-regulated miRNAs under Cr stress, respectively.

### Prediction and annotation of potential targets for Cr-responsive miRNAs

The putative target genes were predicted by the plant small RNA target analysis server (psRNATarget; http://plantgrn.noble.org/psRNATarget/) as described by Dai and Zhao^53^. The criteria for target prediction were based on previous studies suggested by Allen *et al.* and Schwab *et al.*^54^,^55^. To systematically understand the potential functions of miRNA-targeted genes in radish, gene ontology (GO) annotations were assigned using the Blast2GO program^56^. The target sequences were allocated to the corresponding functional categories on the basis of the BLAST searches by GO annotation using default parameters. In addition, KEGG Orthology Based Annotation System (KOBAS 2.0; http://kobas.cbi.pku.edu.cn/home.do) was carried out to predict the biological functions of target genes^57^. To identify the biological pathways affected by Cr stress, KEGG pathway enrichment analysis for the target genes of differentially expressed miRNAs was also performed.

### RT-qPCR validation of miRNAs and their potential targets

Real-time quantitative PCR (RT-qPCR) was performed to experimentally validate the expression patterns of radish miRNAs and their target genes from high-throughput sequencing. RNA and miRNA samples extraction and reverse transcription were carried out according to the previous reports^13^,^45^. Each reaction was performed on a MyiQ Real-Time PCR Detection System (BIO-RAD, USA) with three biological replicates and technological replicates, and the expression levels were normalized to the 5.8S rRNA. The relative expression levels of the miRNAs and targets were calculated using the 2^–ΔΔ*C_T_*^ method^58^. Additionally, the statistical analysis was performed using Duncan’s multiple range test at the *P* < 0.05 level of significance using IBM SPSS Statistics 20.0 (New York, USA) program. The primers for RT-qPCR were designed using Beacon Designer 7.9 software (Supplementary Table S10).

## Additional Information

**How to cite this article**: Liu, W. *et al.* Transcriptome-wide analysis of chromium-stress responsive microRNAs to explore miRNA-mediated regulatory networks in radish (*Raphanus sativus* L.). *Sci. Rep.* **5**, 14024; doi: 10.1038/srep14024 (2015).

## Supplementary Material

Supplementary Information

Supplementary Dataset 1

Supplementary Dataset 2

Supplementary Dataset 3

Supplementary Dataset 4

Supplementary Dataset 5

## Figures and Tables

**Table 1:** Distribution of small RNAs among different categories in radish.

**Category**	**CK**		**Cr200**	
	**Unique sRNAs**	**Total sRNAs**	**Unique sRNAs**	**Total sRNAs**
Total small RNAs	3,360,437 (100%)	18,127,561 (100%)	6,172,992(100%)	19,552,260 (100%)
miRNA	20,156 (0.60%)	1,680,180 (9.27%)	43,980 (0.71%)	2,057,888 (10.53%)
rRNA	68,092 (2.02%)	1,333,046 (7.36%)	101,446 (1.64%)	2,552,322 (13.05%)
snRNA	4,561 (0.14%)	17,456 (0.10%)	10,311 (0.17%)	48,852 (0.25%)
snoRNA	2,399 (0.07%)	4,918 (0.03%)	2,979 (0.05%)	6,596 (0.03%)
tRNA	6,374 (0.19%)	162,311 (0.90%)	12,242 (0.20%)	260,269 (1.33%)
Unannotated	3,258,855 (96.98%)	14,929,650 (82.36%)	6,002,034 (97.23%)	14,626,333 (74.81%)

**Table 2:** Abundance of conserved and non-conserved miRNA families from CK and Cr200 libraries in radish.

**Family**	**Number of members**	**miRNA reads**		**Normalized read count**		**Fold change log_2_(Cr200/CK)**
		**CK**	**Cr200**	**CK**	**Cr200**	
Conserved miRNA						
miR156/157	5	366,511	265,213	20,218.44	13,564.31	−0.58
miR158	2	699,489	1,730,976	38,587.04	88,530.74	1.20
miR159	1	2,645	1,201	145.91	61.43	−1.25
miR160	4	2,652	1,644	146.30	84.08	−0.80
miR161	1	0	151	0.01	7.72	9.59
miR162	1	750	1,438	41.37	73.55	0.83
miR164	2	20,482	36,698	1,129.88	1,876.92	0.73
miR165/166	5	91,078	250,786	5,024.28	12,826.45	1.35
miR167	2	19,807	21,945	1,092.65	1,122.38	0.04
miR168	2	196,680	86,888	10,849.78	4,443.89	−1.29
miR169	4	9,690	4,742	534.55	242.53	−1.14
miR171	1	155	299	8.55	15.29	0.84
miR172	2	1,024	16,755	56.49	856.93	3.92
miR319	2	13,971	8,927	770.70	456.57	−0.76
miR390	2	324	6,464	17.87	330.60	4.21
miR391	2	1,066	2,988	58.81	152.82	1.38
miR393	1	15	13	0.83	0.66	−0.32
miR394	2	201	946	11.09	48.38	2.13
miR395	2	71	247	3.92	12.63	1.69
miR396	2	958	2,301	52.85	117.68	1.16
miR397	1	15,201	927	838.56	47.41	−4.14
miR398	3	1,934	64	106.69	3.27	−5.03
miR399	2	142	87	7.83	4.45	−0.82
miR408	1	266,952	32,197	14,726.31	1,646.72	−3.16
Non-conserved miRNA						
miR400	1	109	84	6.01	4.30	−0.49
miR403	1	1,634	1,825	90.14	93.34	0.05
miR414	1	344	0	18.98	0.01	−10.89
miR415	1	0	229	0.01	11.71	10.19
miR482	1	134	2,254	7.39	115.28	3.96
miR535	2	34	2,034	1.88	104.03	5.79
miR824	2	529	2,477	29.18	126.69	2.12
miR825	1	1,455	1,835	80.26	93.85	0.23
miR827	1	275	509	15.17	26.03	0.78
miR845	2	3,003	5,968	165.66	305.23	0.88
miR854	1	229	387	12.63	19.79	0.65
miR857	1	1,413	240	77.95	12.27	−2.67
miR858	2	23	13	1.27	0.66	−0.93
miR860	1	58	345	3.20	17.65	2.46
miR1885	1	5,417	6,787	298.83	347.12	0.22
miR2111	3	280	104	15.45	5.32	−1.54
miR2118	1	27,628	23,077	1,524.09	1,180.27	−0.37
miR4993	1	0	306	0.01	15.65	10.61
miR5265	1	0	261	0.01	13.35	10.38
miR5293	1	143	551	7.89	28.18	1.84
miR5671	1	0	3,860	0.01	197.42	14.27
miR7767	2	9,153	23,835	504.92	1,219.04	1.27

**Table 3:** Known Cr-responsive miRNAs from CK and Cr200 libraries in radish.

**Family**	**miRNA name**	**miRNA reads**		**Normalized reads**		**Fold change log_2_ (Cr200/CK)**	***P*-value**	**Sig-lable**	**Regulated**
		**CK**	**Cr200**	**CK**	**Cr200**				
miR156/157	miR156a	94,427	59,654	5,209.0295	3,051.0028	−0.77	0		down-regulated
	miR156a-3p	257	0	14.1773	0.0100	−10.47	2.10E-82	**	down-regulated
	miR156f-3p	0	286	0.0100	14.6275	10.51	3.36E-82	^**^	up-regulated
	miR157a	268,528	202,820	14,813.2449	10,373.2254	−0.51	0		down-regulated
	miR157a-3p	3,299	2,453	181.9881	125.4586	−0.54	9.42E-45		down-regulated
miR159	miR159a	2,645	1,201	145.9104	61.4251	−1.25	9.43E-148	^**^	down-regulated
miR160	miR160a	0	411	0.0100	21.0206	11.04	8.12E-118	^**^	up-regulated
	miR160b	80	0	4.4132	0.0100	−8.79	3.66E-26	^**^	down-regulated
	miR160b-3p	2,572	0	141.8834	0.0100	−13.79	0	^**^	down-regulated
	miR160d-3p	0	1,233	0.0100	63.0618	12.62	0	^**^	up-regulated
miR161	miR161	0	151	0.0100	7.7229	9.59	9.81E-44	^**^	up-regulated
miR164	miR164b-3p	130	42	7.1708	2.1481	−1.74	2.24E-13	^**^	down-regulated
miR165/166	miR165a	1,978	11,721	109.1156	599.4703	2.46	0	^**^	up-regulated
	miR165a-3p	173	10,692	9.5435	546.8422	5.84	0	^**^	up-regulated
	miR166e-3p	0	1,029	0.0100	52.6282	12.36	6.65E-294	^**^	up-regulated
	miR166g-3p	285	0	15.7219	0.0100	−10.62	2.66E-91	^**^	down-regulated
miR168	miR168a	195,230	85,348	10,769.7886	4,365.1220	−1.30	0	^**^	down-regulated
miR169	miR169b	884	0	48.7655	0.0100	−12.25	1.24E-281	^**^	down-regulated
	miR169j-3p	0	819	0.0100	41.8877	12.03	4.55E-234	^**^	up-regulated
	miR169m	0	3,923	0.0100	200.6418	14.29	0	^**^	up-regulated
	miR169r-3p	8,806	0	485.7796	0.0100	−15.57	0	^**^	down-regulated
miR172	miR172c	1,024	16,685	56.4886	853.3540	3.92	0	^**^	up-regulated
	miR172e-3p	0	70	0.01	3.5801	8.48	1.18E-20	^**^	up-regulated
miR319	miR319a-3p	710	219	39.1669	11.2008	−1.81	1.17E-69	^**^	down-regulated
	miR319b-5p	13,261	8,708	731.538	445.3705	−0.72	2.73E-290		down-regulated
miR390	miR390a	314	6,289	17.3217	321.6508	4.21	0	^**^	up-regulated
	miR390a-3p	10	175	0.5516	8.9504	4.02	1.07E-37	^**^	up-regulated
miR391	miR391-3p	0	21	0.0100	1.0740	6.75	1.08E-06	^**^	up-regulated
miR394	miR394a	14	613	0.7723	31.3519	5.34	1.21E-151	^**^	up-regulated
miR395	miR395a	71	0	3.9167	0.0100	−8.61	2.65E-23	^**^	down-regulated
	miR395b	0	247	0.0100	12.6328	10.30	4.35E-71	^**^	up-regulated
miR397	miR397a	15,201	927	838.5574	47.4114	−4.14	0	^**^	down-regulated
miR398	miR398b-3p	133	0	7.3369	0.0100	−9.52	5.28E-43	^**^	down-regulated
	miR398b-5p	0	64	0.0100	3.2733	8.35	6.03E-19	^**^	up-regulated
	miR398c-5p	1,801	0	99.3515	0.0100	−13.28	0	^**^	down-regulated
miR399	miR399b	0	87	0.0100	4.4496	8.80	1.69E-25	^**^	up-regulated
	miR399h-5p	142	0	7.8334	0.0100	−9.61	7.29E-46	^**^	down-regulated
miR408	miR408-5p	266,952	32,197	14,726.3054	1,646.7150	−3.16	0	^**^	down-regulated
miR414	miR414	344	0	18.9766	0.0100	−10.89	4.76E-110	^**^	down-regulated
miR415	miR415	0	229	0.0100	11.71	10.19	5.85E-66	^**^	up-regulated
miR482	miR482a-5p	134	2,254	7.3921	115.2808	3.96	0	^**^	up-regulated
miR535	miR535b	0	2,034	0.01	104.0289	13.34	0	^**^	up-regulated
	miR535d	34	0	1.8756	0.0100	−7.55	1.51E-11	^**^	down-regulated
miR824	miR824-3p	109	1,565	6.0129	80.0419	3.73	1.57E-307	^**^	up-regulated
miR845	miR845d	1,282	74	70.7148	3.7847	−4.22	9.51E-306	^**^	down-regulated
miR857	miR857	1,413	240	77.9407	12.2748	−2.67	4.08E-222	^**^	down-regulated
miR860	miR860	58	345	3.1995	17.645	2.46	2.01E-46	^**^	up-regulated
miR2111	miR2111a-3p	140	0	7.7230	0.0100	−9.59	3.15E-45	^**^	down-regulated
	miR2111a-5p	140	61	7.7230	3.1198	−1.31	7.17E-10	^**^	down-regulated
	miR2111b-3p	0	43	0.0100	2.1992	7.78	5.81E-13	^**^	up-regulated
miR4993	miR4993	0	306	0.0100	15.6504	10.61	6.72E-88	^**^	up-regulated
miR5265	miR5265	0	261	0.0100	13.3488	10.38	4.46E-75	^**^	up-regulated
miR5293	miR5293	143	771	7.8885	39.4328	2.29	5.15E-51	^**^	up-regulated
miR5671	miR5671	0	3,860	0.0100	197.42	14.27	0	^**^	up-regulated

^**^indicates significant differences in expression between two libraries at *P* < 0.01.

**Table 4:** The novel Cr-responsive miRNAs from CK and Cr200 libraries in radish.

**miRnNA**	**Sequence (5′-3′)**	**CK**		**Cr200**		**Fold change log_2_(Cr200/CK)**	***P*-value**	**Sig-lable**	**Regulated**
		**Count**	**Normalized**	**Count**	**Normalized**				
rsa-miRn-1	AAAUCAUACUUUCAUUGAUA	277	15.2806	0	0.0100	−10.58	9.26E-89	**	down-regulated
rsa-miRn-2	UGGAUAUGAUGUAGUUGAUCCGA	1,275	70.3349	0	0.0100	−12.78	0	^**^	down-regulated
rsa-miRn-3	AGCAAACGAGAAUUGAACGGA	419	23.1140	192	9.8198	−1.24	1.78E-24	^**^	down-regulated
rsa-miRn-10	UGGAUGUAGAGGCAUUUCUUC	79	4.3580	0	0.0100	−8.77	7.60E-26	^**^	down-regulated
rsa-miRn-12	ACATTGGACTACATATATTAC	81	4.4683	935	47.8206	3.42	2.09E-171	^**^	up-regulated
rsa-miRn-14	CGUACGAGGAGCCAAGCAUGA	833	45.9521	0	0.0100	−12.17	1.99E-265	^**^	down-regulated
rsa-miRn-19	GCUCAAGAAAGCUGUGGGAAA	155	8.5505	0	0.0100	−9.74	5.40E-50	^**^	down-regulated
rsa-miRn-20	UCCCUUUGGAUGUCGUCUUGUG	20	1.1033	0	0.0100	−6.79	4.25E-07	^**^	down-regulated
rsa-miRn-23	UCAAUGAAAGGUAUGAUUCCC	0	0.0100	377	19.2817	10.91	3.96E-108	^**^	up-regulated
rsa-miRn-28	GGUCUUUGGGAGUUGGAUUAUCAUC	0	0.0100	856	43.7801	12.10	1.31E-244	^**^	up-regulated
rsa-miRn-44	CGGUGGUGGAGGUGGAGGCGG	0	0.0100	46	2.3527	7.88	8.12E-14	^**^	up-regulated
rsa-miRn-45	UCAGCCGAGGUUCCAUUACCAC	0	0.0100	206	10.5359	10.04	2.09E-59	^**^	up-regulated
rsa-miRn-46	UGUUUUGUGCGUGAAUCUAAUU	0	0.0100	48	2.4550	7.94	2.18E-14	^**^	up-regulated
rsa-miRn-47	CGAAGUGACUUAUAAUGAUCU	0	0.0100	32	1.6366	7.35	7.91E-10	^**^	up-regulated
rsa-miRn-54	AGGAUUGAGUCUAGAAGCAUA	0	0.0100	125	6.3931	9.32	2.51E-36	^**^	up-regulated
rsa-miRn-55	UGGAUACAGUGAUGAUGACGAU	0	0.0100	20	1.0229	6.68	2.08E-06	^**^	up-regulated

^**^indicates significant differences in expression between two libraries at *P* < 0.01.

**Table 5:** Identified candidate targets for known and novel Cr-responsive miRNAs in radish.

**miRNA**	**Target gene No.**	**Target gene description**	**Target annotation**	**At locus**
miR156/157	Rsa#S43017568	Squamosa promoter-binding-like protein 3	*SPL3*	AT2G33810
	CL754.Contig1	Squamosa promoter-binding-like protein 15	*SPL15*	AT3G57920
	CL1121.Contig1	Squamosa promoter-binding-like protein 6	*SPL6*	AT1G69170
	CL7151.Contig1	Squamosa promoter-binding-like protein 13	*SPL13*	AT5G50670
	Unigene34872	Squamosa promoter-binding-like protein 9	*SPL9*	AT2G42200
miR159	Rsa#S42037487	Myb domain protein 101	*MYB101*	AT2G32460
	Rsa#S41979156	Putative transcription factor SPL	*SPL*	AT4G27330
	Unigene16165	Myb domain protein 104	*MYB104*	AT2G26950
miR160	Rsa#S42581764	Auxin response factor 16	*ARF16*	AT4G30080
	Unigene495	Aluminum-activated malate transporter 9	*ALMT9*	AT3G18440
miR164	Unigene26020	Transcription factor NAC1	*NAC1*	AT1G56010
	Rsa#S43010415	Ethylene-responsive transcription factor ERF073	*HRE1*	AT1G72360
miR165	CL6137.Contig2	Homeobox-leucine zipper protein ATHB-14	*PHB*	AT2G34710
miR166	Unigene28911	Homeobox-leucine zipper protein ATHB-9	*PHV*	AT1G30490
miR172	CL658.Contig1	Transcription factor IIIA	*TFIIIA*	AT1G72050
miR391	Unigene27169	Calcium-transporting ATPase 10	*ACA10*	AT4G29900
miR397	Unigene3675	Laccase 17	*LAC17*	AT5G60020
miR415	CL13325.Contig1	Ethylene-responsive transcription factor ERF116	*ERF116*	AT1G25470
miR824	CL1245.Contig2	Agamous-like MADS-box protein AGL16	*AGL16*	AT3G57230
miR825	CL538.Contig1	TIR-NBS-LRR class disease resistance protein		AT1G63880
	Unigene16565	TIR-NBS-LRR class disease resistance protein		AT1G63880
miR854	CL5587.Contig1	WRKY DNA-binding protein 26	*WRKY26*	AT5G07100
miR1885	CL10476.Contig1	TIR-NBS-LRR class disease resistance protein		AT1G63880
miR4993	CL4937.Contig1	DEAD-box ATP-dependent RNA helicase 52		AT3G58570
	CL12544.Contig1	Heavy metal transport/detoxification domain-containing protein		AT5G24580
	CL2080.Contig2	Translation initiation factor IF-3		AT4G30690
miR5265	Unigene11660	Metal-nicotianamine transporter YSL1	*YSL1*	AT4G24120
miR5671	Unigene10987	Putative copper-transporting ATPase HMA5	*HMA5*	AT1G63440
miR5293	Rsa#S42010504	Transcription factor TCP15	*TCP15*	AT1G69690
	CL2525.Contig3	HSP90-like protein GRP94	*SHD*	AT4G24190
	CL3795.Contig3	cytochrome P450 90A1	*CPD*	AT5G05690
	Unigene10271	Transcription factor TCP21	*TCP21*	AT5G08330
	CL2041.Contig2	Putative galacturonosyltransferase-like 7	*GATL7*	AT3G62660
	CL10461.Contig2	Mitogen-activated protein kinase kinase kinase 1	*MEKK1*	AT4G08500
	Rsa#S42588632	Transcription factor TCP6	*TCP6*	AT5G41030
	CL8348.Contig1	Ethylene-responsive transcription factor ERF094	*ORA59*	AT1G06160
miR7767	CL243.Contig2	ABC transporter G family member 33	*PDR5*	AT2G37280
rsa-miRn16	CL443.Contig1	Putative WRKY transcription factor 42	*WRKY42*	AT4G04450
rsa-miRn20	Rsa#S42580584	Auxin signaling F-box 3 protein	*AFB3*	AT1G12820
	CL4893.Contig2	Protein TRANSPORT INHIBITOR RESPONSE 1	*TIR1*	AT3G62980
rsa-miRn28	CL335.Contig2	Nicotinate phosphoribosyltransferase 1	*NAPRT1*	AT4G36940
rsa-miRn39	CL11639.Contig2	Heat shock protein 81-2	*HSP81-2*	AT5G56030
rsa-miRn44	Unigene1136	proline-rich family protein		AT1G02405
	CL11490.Contig1	Auxin response factor 2	*ARF2*	AT5G62000
	Unigene2794	Heavy-metal-associated domain-containing protein		AT1G57780
	Rsa#S42032440	Transcription factor bHLH147	*bHLH147*	AT3G17100
	Unigene14136	Heavy metal transport/detoxification domain-containing protein		AT5G24580
	FD958446	Thioredoxin-like protein CITRX	*TRXz*	AT3G06730
	CL6144.Contig1	Ethylene-responsive transcription factor 7	*ERF7*	AT3G20310
	CL1166.Contig1	bZIP protein	*bZIP*	AT5G04840
rsa-miRn55	CL5442.Contig2	ABC transporter D family member 1	*PXA1*	AT4G39850
