# Supplementary material for: Transcriptome-wide analysis of chromium-stress responsive microRNAs to explore miRNA-mediated regulatory networks in radish (Raphanus sativus L.)
Source: Sci Rep. 2015 Sep 11;5:14024. doi: 10.1038/srep14024 (PMC4566140; doi:10.1038/srep14024)
Supplement: Supplementary Information [file srep14024-s1.pdf]

Supplementary information

Transcriptome-wide analysis of chromium-stress responsive microRNAs to explore miRNA-mediated regulatory networks in radish (*Raphanus sativus* L.)

Wei Liu, Liang Xu, Yan Wang, Hong Shen, Xianwen Zhu, Keyun Zhang, Yinglong Chen, Rugang Yu, Cecilia Limera, Liwang Liu*

Supplementary Figures

Supplementary Fig. S1: Venn diagrams for analysis of total (A) and unique (B) sRNAs between CK and Cr200 libraries from radish roots.

Supplementary Fig. S2: First nucleotide bias of novel miRNA candidates in radish CK (A) library and Cr200 (B) library.

Supplementary Fig. S3: Secondary structure of novel *Raphanus sativus* miRNA precursor. Mature miRNA is in red and miRNA* is in blue. Precursor secondary structure was produced using the mfold software (http://mfold.bioinfo.rpi.edu/). (“.” represents base mismatches, “(” represents base matches).

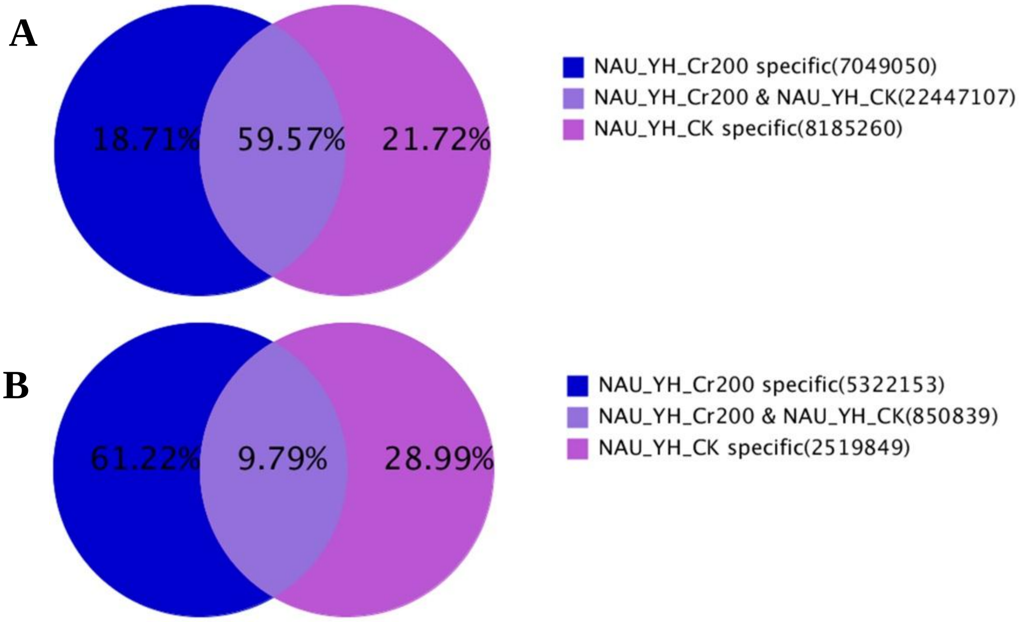

Supplementary Fig. S1 Venn diagrams for analysis of total (A) and unique (B) sRNAs between CK and Cr200 libraries from radish roots.

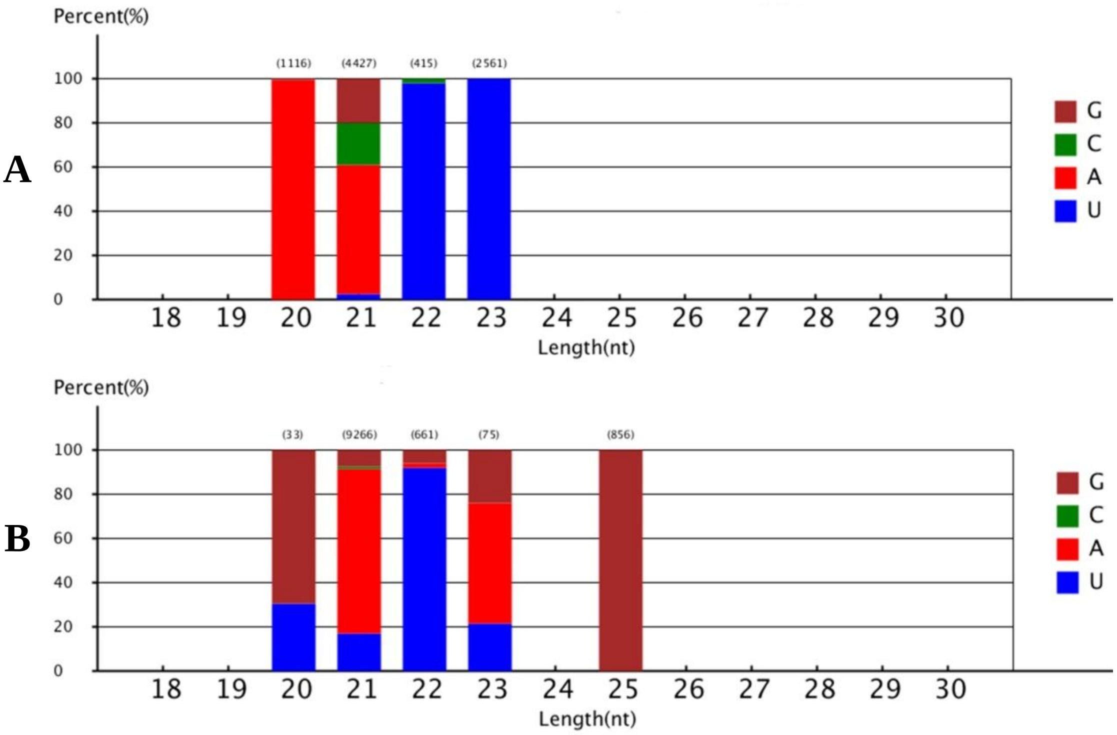

Supplementary Fig. S2 First nucleotide bias of novel miRNA candidates in radish CK (A) library and Cr200 (B) library.

Supplementary Fig. S3 Secondary structure of novel *Raphanus sativus* miRNA precursor. Mature miRNA is in red and miRNA* is in blue. Precursor secondary structure was produced using the mfold software (http://mfold.bioinfo.rpi.edu/). (“.” represents base mismatches, “(” represents base matches).

rsa-miRn1a

NAU_YH_CK-m0001 CL11776.Contig1_NAU-YH:1245:1428:+ 184(nt) -68.61(kcal/mol)

CAAAGAAAAGAAATCATACTTTCATTGATAATGAAATCAATAGAGAGAATGATAATGAGCTCTTACATGCAATTCAAAAATATAGTATCTCTTAGATCCTCTAATTGCTTGTAAGAGTTGATGACCAATGGG
TTATCCTCTATTGATCTTATCATCAATGAAAGGTATGATTCCCTTTCTTTGA
(((((((.(.((((((((((((((((((.((((.((((((((((............(((((((((.(((((.......((.((.(((.....))).)).))))))).))))))))))(((((((((....))
))))))))))))))).)))).)))))))))).)))))))).).)))))))).
**********AAATCATACTTTCATTGATA**
****
****
**********************TCAATGAAAGGTATGATTCCC*********

rsa-miRn1b

NAU_YH_CK-m0002 CL11776.Contig1_NAU-YH:672:856:- 185(nt) -74.31(kcal/mol)

CAAAGAAAAGAAATCATACTTTCATTGATAATGAAATCAATAGAGAGAATGATAATGAGCTCTTACATGCAATTCAAAAATATAGTATCTCTTAGATCCTCTAATTGCTTGTAAGAGTTGATGACCAAATGG
GTTATCCTCTATTGATTTCATCATCAATGAAAGGTATGATTCCCTTTCTTTGA
(((((((.(.((((((((((((((((((.(((((((((((((((............(((((((((.(((((.......((.((.(((.....))).)).))))))).))))))))))(((((((((.....)
))))))))))))))))))))).)))))))))).)))))))).).)))))))).
**********AAATCATACTTTCATTGATA**
****
****
***********************TCAATGAAAGGTATGATTCCC*********

rsa-miRn12

NAU_YH_Cr200-m0034 Unigene27956_NAU-YH:130:246:- 117(nt) -66.00(kcal/mol)

TTGGTCAAGTGTAATGTATGTAGTCCAATCTATTGAAGTACTAGCACACCATCTCTAGTGAGGTGATGTGGTTTGAGTAGTTCAATACATTGGACTACATATATTACATTTGATCAA
.((((((((((((((((((((((((((((.(((((((.(((((.((((.((((((....)))))).)))).)...)))).))))))).)))))))))))))))))))))))))))).
************AATGTATGTAGTCCAATCTAT**
ACATTGGACTACATATATTAC********

rsa-miRn23a

NAU_YH_Cr200-m0002 CL11776.Contig1_NAU-YH:1245:1428:+ 184(nt) -68.61(kcal/mol)

CAAAGAAAAGAAATCATACTTTCATTGATAATGAAATCAATAGAGAGAATGATAATGAGCTCTTACATGCAATTCAAAAATATAGTATCTCTTAGATCCTCTAATTGCTTGTAAGAGTTGATGACCAATGGG
TTATCCTCTATTGATCTTATCATCAATGAAAGGTATGATTCCCTTTCTTTGA NAU_YH_Cr200-m0002 761
((((((((.(.((((((((((((((((((.((((.((((((((((............((((((((((.(((((.......((.((.(((.....))).)).))))))).))))))))))(((((((....))
))))))))))))))).)))).)))))))))).)))))))).).)))))))).
**********AAATCATACTTTCATTGATA**
****
****
**********************TCAATGAAAGGTATGATTCCC*********

rsa-miRn23b

NAU_YH_Cr200-m0003 CL11776.Contig1_NAU-YH:672:856:- 185(nt) -74.31(kcal/mol)

CAAAGAAAAGAAATCATACTTTCATTGATAATGAAATCAATAGAGAGAATGATAATGAGCTCTTACATGCAATTCAAAAATATAGTATCTCTTAGATCCTCTAATTGCTTGTAAGAGTTGATGACCAAATGG
GTTATCCTCTATTGATTTCATCATCAATGAAAGGTATGATTCCCTTTCTTTGA
((((((((.(.((((((((((((((((((.(((((((((((((((............((((((((((.(((((.......((.((.(((.....))).)).))))))).))))))))))(((((((.....)
))))))))))))))))))))).)))))))))).)))))))).).)))))))).
**********AAATCATACTTTCATTGATA**
****
****
***********************TCAATGAAAGGTATGATTCCC*********

rsa-miRn28

NAU_YH_Cr200-m0011 CL335.Contig2_NAU-YH:1342:1443:- 102(nt) -47.20(kcal/mol)

GTTGTGATGATAATCTGACTCCAAGACCGAAACAACAACAAGTTCTTATCATTTGTTTGATGGTATTGATTTGGTCTTTGGGAGTTGGATTATCATCACAAG
.((((((((((((((..(((((.((((((((.(((...........(((((((.....)))))))))).))))))))...)))))..))))))))))))))).
*******TGATAATCTGACTCCAAGACCGA**
GGTCTTTGGGAGTTGGATTATCATC***

Supplementary Tables

Supplementary Table S1: Summary of common and specific sequences between CK and Cr200 sRNA libraries.

Supplementary Table S2: Summary of clean data produced from CK and Cr200 sRNA libraries of radish roots.

Supplementary Table S3: Statistical analysis of sequencing reads from the CK and Cr200 sRNA libraries in radish.

Supplementary Table S4: Detailed information of the known miRNAs identified from radish roots.

Supplementary Table S5: Detailed information of novel candidate miRNAs identified from radish roots (See Excel file).

Supplementary Table S6: Details of potential targets for the differentially expressed miRNAs from blast2GO program (See Excel file).

Supplementary Table S7: KEGG pathway enrichment analysis for the targets of differentially expressed miRNAs (See Excel file).

Supplementary Table S8: Putative known and novel miRNA target genes in radish (See Excel file).

Supplementary Table S9: Details of unclassified miRNA target genes in radish (See Excel file).

Supplementary Table S10: Primers of validated miRNAs and target genes for RT-qPCR.

Table S1 Summary of common and specific sequences between CK and Cr200 sRNA libraries.

Class	Unique sRNAs	Percentage	Total sRNAs	Percentage
Total_sRNAs	8,692,841	100.00%	37,681,417	100.00%
CK & Cr200	850,839	9.79%	22,447,107	59.57%
CK_specific	2,519,849	28.99%	8,185,260	21.72%
Cr200_specific	5,322,153	61.22%	7,049,050	18.71%

Table S2 Summary of clean data produced from CK and Cr200 sRNA libraries of radish roots.

Category	CK		Cr200	
	**Count**	**Percentage**	**Count**	**Percentage**
Raw_reads	18,473,237		20,530,194	
High_quality	18,299,929	100%	20,427,316	100%
3' adapter_null	2,265	0.01%	13,301	0.07%
Insert_null	1,699	0.01%	3,195	0.02%
5' adapter_contaminants	94,374	0.52%	168,087	0.82%
Smaller_than_18nt	72,956	0.40%	687,409	3.37%
Poly (A)	1,074	0.01%	3,064	0.01%
Clean_reads	18,127,561	99.06%	19,552,260	95.72%

Table S3 Statistical analysis of sequencing reads from the CK and Cr200 sRNA libraries in radish.

	Total sRNAs	Unique sRNAs
**CK**		
Raw reads	18,473,237	
Clean reads	18,127,561	3,360,437
Mapped to genomic	8,814,297	582,092
Match known miRNAs	1,680,180	20,156
Unannotated sRNAs	14,929,650	3,258,855
**Cr200**		
Raw reads	20,530,194	
Clean reads	19,552,260	6,172,992
Mapped to genomic	6,236,509	742,492
Match known miRNAs	2,057,888	43,980
Unannotated sRNAs	14,626,333	6,002,034

Table S4 Detailed information of the known miRNAs identified from radish roots.

Family	Number	Members	Sequence (5' to 3')	miRNA reads		Normalized read count		Fold change log_2 (Cr200/CK)
				CK	Cr200	CK	Cr200	
**Conserved miRNA**								
miR156/157	5	miR156a	UGACAGAAGAGAGUGAGCAC	94,427	59,654	5,209.0295	3,051.0028	-0.77
		miR156a-3p	UGCUCACGGCUCUUUCUGUCAGU	257	0	14.1773	0.0100	-10.47
		miR156f-3p	GCUCACUUCUCUUUCUGUCAGA	0	286	0.0100	14.6275	10.51
		miR157a	UUGACAGAAGAUAGAGAGCAC	268,528	202,820	14,813.2449	10,373.2254	-0.51
		miR157a-3p	GCUCUCUAGCCUUCUGUCAUC	94,427	59,654	5,209.0295	3,051.0028	-0.54
miR158	2	miR158a	UCCCAAACGUAGACAAAGCA	348,667	873,736	19,234.0823	44,687.2126	1.22
		miR158b-3p	UCCCAAACGUAGACAAAGC	350,822	857,240	19,352.9620	43,843.5250	1.18
miR159	1	miR159a	UUUGGAUUGAAGGGAGCUCUA	2,645	1,201	145.9104	61.4251	-1.25
miR160	4	miR160a	UGCCUGGCUCCCUGUAUGCCA	0	411	0.0100	21.0206	11.04
		miR160b	AUGCCUGGCUCCCUGUAUGCC	80	0	4.4132	0.0100	-8.79
		miR160b-3p	GCGUAUGAGGAGCCAUGCAUA	2,572	0	141.8834	0.0100	-13.79
		miR160d-3p	CGUACGAGGAGCCAAGCAUGA	0	1,233	0.0100	63.0618	12.62
miR161	1	miR161	UCAAUGCACUGAAAGUGACUA	0	151	0.0100	7.7229	9.59
miR162	1	miR162a	UCGAUAAACCUCUGCAUCCAG	750	1,438	41.3735	73.5465	0.83
miR164	2	miR164a	UGGAGAAGCAGGGCACGUGCA	20,352	36,656	1,122.7103	1,874.7705	0.74
		miR164b-3p	CAUGUGCCCAUCUUCCCCAUC	130	42	7.1714	2.1481	-1.74
miR165/166	5	miR165a	UCGGACCAGGCUUCAUCCCCC	1,978	11,721	109.1156	599.4703	2.46
		miR165a-3p	UCGGACCAGGCUUCAUCCCC	173	10,692	9.5435	546.8422	5.84
		miR166a	UCGGACCAGGCUUCAUUCCCC	88,642	227,344	4,889.9022	11,627.5050	1.25
		miR166e-3p	UCGAACCAGGCUUCAUUCCCC	0	1,029	0.0100	52.6282	12.36
		miR166g-3p	UCGGACCAGGCUUCAUUCCUC	285	0	15.7219	0.0100	-10.62
miR167	2	miR167f-3p	AGAUCAUGUUUGCAGUUUCACC	3,906	3,137	215.4730	160.4418	-0.43
		miR167f-5p	UGAAGCUGCCAGCGUGAUCUU	15,901	18,808	877.1726	961.9348	0.13
miR168	2	miR168a	UCGCUUGGUGCAGGUCGGGAC	195,230	85,348	10,769.7886	4,365.1220	-1.30
		miR168a-3p	GAUCCCGCCUUGUAUCAAGUGAAU	1,450	1,540	79.9887	78.7633	-0.02
miR169	4	miR169b	CAGCCAAGGAUGACUUGCCGG	884	0	48.7655	0.0100	-12.25
		miR169j-3p	GGCAGUCUCCUUGGCUAUC	0	819	0.0100	41.8877	12.03
		miR169m	UGAGCCAAAGAUGACUUGCCG	0	3,923	0.0100	200.6418	14.29
		miR169r-3p	GGCACGUGUCGUUGGCUAA	8,806	0	485.7796	0.0100	-15.57
miR171	1	miR171a	UUGAGCCGUGCCAAUAUCUCU	155	299	8.5505	15.2923	0.84

miR172	2	miR172c	AGAAUCUUGAUGAUGCUGCAG	1,024	16,685	56.4886	853.3540	3.92
		miR172e-3p	GAAUCUUAUGGACUGUUGCAU	0	70	0.0100	3.5801	8.48
miR319	2	miR319a-3p	UUGGACUGAAGGGAGCUCCC	710	219	39.1669	11.2008	-1.81
		miR319b-5p	GAGCUUUCUUCGGUCCACUC	13,261	8,708	731.5380	445.3705	-0.72
miR390	2	miR390a	AAGCUCAGGAGGGAUAGCGCC	314	6,289	17.3217	321.6508	4.21
		miR390a-3p	CGCUGUCCAUCCUGAGUUUCA	10	175	0.5516	8.9504	4.02
miR391	2	miR391	UUCGCAGGAGAGAUAGCGCCA	1,066	2,967	58.8055	151.7472	1.37
		miR391-3p	ACGGUAUCUCCCCUACGUAGC	0	21	0.0100	1.0740	6.75
miR393	1	miR393b-3p	AUCAUGCGAUCUCUUCGGAUU	15	13	0.8275	0.6649	-0.32
miR394	2	miR394a	UUGGCAUUCUGUCCACCUCC	14	613	0.7723	31.3519	5.34
		miR394b-3p	AGGUGGUCAUACUGUCAACA	187	333	10.3158	17.0313	0.72
miR395	2	miR395a	CUGAAGUGUUUGGGGGAACUC	71	0	3.9167	0.0100	-8.61
		miR395b	CUGAAGUGUUUGGGGGGACUC	0	247	0.0100	12.6328	10.30
miR396	2	miR396a	UUCCACAGCUUUCUUGAACUG	454	1,441	25.0447	73.6999	1.56
		miR396b-3p	GCUCAAGAAAGCUGUGGGAAA	504	860	27.8030	43.9847	0.66
miR397	1	miR397a	UCAUUGAGUGCAGCGUUGAUGU	15,201	927	838.5574	47.4114	-4.14
miR398	3	miR398b-3p	UUUGUGUUCUCAGGUCACCCC	133	0	7.3369	0.0100	-9.52
		miR398b-5p	GGGUUGACAUGAGAACACAUG	0	64	0.0100	3.2733	8.35
		miR398c-5p	GGGUCGAUAUGAGAACACAUG	1,801	0	99.3515	0.0100	-13.28
miR399	2	miR399b	UGCCAAAGGAGAGUUGCCCUG	0	87	0.0100	4.4496	8.80
		miR399h-5p	GGGCAAGAUCUCUAUUGGCAGG	142	0	7.8334	0.0100	-9.61
miR408	1	miR408-5p	ACAGGGAACAAGCAGAGCAUG	266,952	32,197	14,726.3054	1,646.7150	-3.16
**Non-conserved miRNA**								
miR400	1	miR400	UAUGAGAGUAUUAUAAGUCAC	109	84	6.0129	4.2962	-0.48
miR403	1	miR403	UUAGAUUCACGCACAAACUCG	1,634	1,825	90.1390	93.3396	0.05
miR414	1	miR414	UCAUCUUCUUCCACCAUGUCA	344	0	18.9766	0.0100	-10.89
miR415	1	miR415	GCGCAGAACAGAGAACAGAGCAGU	0	229	0.01	11.71	10.19
miR482	1	miR482a-5p	AGAGAUGAUGUGGGCAAUGGGCUG	134	2,254	7.3921	115.2808	3.96
miR535	2	miR535b	UGAACAAGGAAAGAGAGCAGG	0	2,034	0.0100	104.0289	13.34
		miR535d	GACGACGACGGAGCAGAGCACGAC	34	0	1.8756	0.01	-7.55
miR824	2	miR824	UAGACCAUUUGUGAGAAGGGA	420	912	23.1691	46.6442	1.01
		miR824-3p	CCUUCUCGUCGAUGGUCUAGA	109	1,565	6.0129	80.0419	3.73
miR825	1	miR825-5p	UCCAGACACCAGCUUGAAGAAG	1,455	1,835	80.2645	93.8510	0.23
miR827	1	miR827a	UUAGAUGACCAUCAACAAAUA	275	509	15.1703	26.0328	0.78
miR845	2	miR845a-3p	CUGGCUCUGAUACCAUGUAGAU	1,721	5,894	94.9383	301.4485	1.67
		miR845d	UGGCUCUGAUACCAACUGAUGUAG	1,282	74	70.7210	3.7847	-4.22

miR854	1	miR854	AUGAGGAGAGUGAGGAAGGUG	229	387	12.6327	19.7931	0.65
miR857	1	miR857	CAUUUUGUAUGUUGAAGGUGU	1,413	240	77.9476	12.2748	-2.67
miR858	2	miR858-3p	GUCGGUCAGACAACGAAGAUA	17	0	0.9378	0.0100	-6.55
		miR858a	UUUCGUUGUCUGUUCGACCUU	6	13	0.3310	0.6649	1.01
miR860	1	miR860	UCAAUACAUUGGACUACAUAU	58	345	3.1995	17.6450	2.46
miR1885	1	miR1885b	UACAUCUUCUCCGCGGAAGCUC	5,417	6,787	298.8267	347.1210	0.22
miR2111	3	miR2111a-3p	GUCCUCGGGAUGCGGAUUACC	140	0	7.7230	0.0100	-9.59
		miR2111a-5p	UAAUCUGCAUCCUGAGGUUUA	140	61	7.7230	3.1198	-1.31
		miR2111b-3p	AUCCUCGGGAUACAGAUUACC	0	43	0.0100	2.1992	7.78
miR2118	1	miR2118-5p	GUCGAUGGAACAAUGUAGGCAAGG	27,628	23,077	1,524.0881	1,180.2728	-0.37
miR4993	1	miR4993	GAGGUGGAGGCGGUGGAGGUG	0	306	0.0100	15.6504	10.61
miR5265	1	miR5265	AAGUGAUAGUUGGAUAUUA	0	261	0.0100	13.3488	10.38
miR5671	1	miR5671	CAUGGUGGUAACGGGUGAC	0	3860	0.01	197.42	14.27
miR5293	1	miR5293	AGAGGAAGUGGAAGAAGAAGA	143	551	7.8885	28.1809	2.29
miR7767	2	miR7767-3p	AUGGAGAAGAAGCUUGAUGGU	60	123	3.3099	6.2908	0.93
		miR7767-5p	CACCAAGCUGAAGAUCUCC	9,093	23,712	501.6119	1,212.7498	1.27

Table S10 Primers of validated miRNAs and target genes for RT-qPCR.

**miRNA & targets**	**primer sequence (5'-3')**
miR156a	TGACAGAAGAGAGTGAGCAC
miR156a-3p	TGCTCACGGCTCTTTCTGTCAGT
miR159a	TTTGGATTGAAGGGAGCTCTA
miR160a	TGCCTGGCTCCCTGTATGCCA
miR160b-3p	GCGTATGAGGAGCCATGCATA
miR160d-3p	CGTACGAGGAGCCAAGCATGA
miR165a	TCGGACCAGGCTTCATCCCCC
miR165a-3p	TCGGACCAGGCTTCCCCCC
miR166e-3p	TCGAACCAGGCTTCATTCCCC
miR168a	TCGCTTGGTGCAGGTCGGGAC
miR169b	CAGCCAAGGATGACTTGCCGG
miR169j-3p	GGCAGTCTCCTTGGCTATC
miR169r-3p	AAGGCACGTGTCGTTGGCTAA
miR319b-5p	GAGCTTTCTTCGGTCCACTC
miR390a	AAGCTCAGGAGGGATAGCGCC
miR398c-5p	GGGTCGATATGAGAACACATG
miR408-5p	ACAGGGAACAAGCAGAGCATG
miR164b-3p	CATGTGCCCATCTTCCCCATC
miR4993	GAGGTGGAGGCGGTGGAGGTG
miR5293	AGAGGAAGTGGAAGAAGAAGA
miR5671	CATGGTGGTAACGGGTGAC
rsa-miRn2	TGGATATGATGTAGTTGATCCGA
rsa-miRn4	AGGCCAAGGAAGTTTGAGGCA
rsa-miRn14	CGTACGAGGAGCCAAGCATGA
rsa-miRn19	GCTCAAGAAAGCTGTGGGAAA
rsa-miRn44	CGGTGGTGGAGGTGGAGGCGG
rsa-miRn55	TGGATACAGTGATGATGACGAT
Reverse miRNA	GCGAGCACAGAATTAATACGAC
PHB-F	GGTTCCATCATTCACATTG
PHB-F	ACTTCTCCACTATTCTCTTG
TCP15-F	CGAGTCACATTCTTCTTCT
TCP15-R	CTGCGTATAATCACCACTA
HMA5-F	GCCTGTAGTTCCTTGATT
HMA5-R	TGCTAGAGAAGTCGGTAT
PXA1-F	AAGAGGATAGCAGTGAGA
PXA1-R	TGGACATAGTGATGATGAC
bHLH147-F	CTCTCCACATCCTTTCTG
bHLH147-R	GTCTGATTCGAGTCCATTA
